# Ebola virus disease complicated with viral interstitial pneumonia: a case report

**DOI:** 10.1186/s12879-015-1169-4

**Published:** 2015-10-16

**Authors:** Nicola Petrosillo, Emanuele Nicastri, Simone Lanini, Maria Rosaria Capobianchi, Antonino Di Caro, Mario Antonini, Vincenzo Puro, Francesco Nicola Lauria, Nakono Shindo, Nicola Magrini, Gary P. Kobinger, Giuseppe Ippolito, INMI EBOV Team

**Affiliations:** National Institute for Infectious Diseases Lazzaro Spallanzani-INMI IRCCS, Rome, Italy; Pandemic and Epidemic Diseases Department, World Health Organization, Geneva, Switzerland; Essential Medicines and Health Products Department, World Health Organization, Geneva, Switzerland; Special Pathogens Program, National Microbiology Laboratory, Public Health Agency of Canada, Winnipeg, MB Canada

**Keywords:** Hemorrhagic fever, Ebola, Pneumonia, viral, Malaria, vivax, Immunization, passive, Antibodies, monoclonal, Favipiravir, Fluid therapy, Life support care, Hospitals, isolation

## Abstract

**Background:**

In the current Ebola epidemic in Western Africa, many healthcare workers have become infected. Some of these have been medically evacuated to hospitals in Europe and the USA. These clinical experiences provide unique insights into the course of Ebola virus disease under optimized condition within high level isolation units.

**Case presentation:**

A 50-year-old Caucasian male physician contracted Ebola virus diseases in Sierra Leone and was medically evacuated to Italy. Few days after the admission the course of the illness was characterized by severe gastro-intestinal symptoms followed by respiratory failure, accompanied by pulmonary infiltration and high Ebola viral load in the bronchial aspirate and Plasmodium vivax co-infection. The patient received experimental antiviral therapy with favipiravir, convalescent plasma and ZMAb. Ebola viral load started to steadily decrease in the blood after ZMAb administration and became undetectable by day 19 after admission, while it persisted longer in urine samples. No temporal association was observed between viral load decay in plasma and administration of favipiravir. The patient completely recovered and was discharged 39 days after admission.

**Conclusions:**

This is the first case of Ebola-related interstitial pneumonia documented by molecular testing of lung fluid specimens. This reports underlines the pivotal role of fluid replacement and advanced life support with mechanical ventilation in the management of patients with Ebola virus diseases respiratory failure. Beside our finding indicates a close temporal association between administration of cZMAb and Ebola virus clearance from blood.

**Electronic supplementary material:**

The online version of this article (doi:10.1186/s12879-015-1169-4) contains supplementary material, which is available to authorized users.

## Background

Since December 2013, the Ebola virus (EBOV) Makona variant has been gripping Western Africa [1]. Over this time the epidemic has exponentially grown and has moved to Europe and North America, with several imported cases and even few clusters of local transmission [2, 3]. An unprecedented number of healthcare workers (HCWs) from different countries have been infected, some of whom died [4]. In total 26 cases of Ebola virus diseases (EVD), 4 of which fatal [5], have been treated in Europe [6–8] and North America [9, 10] since the beginning of the outbreak.

Hereby we describe the successful management of a 50-year-old Italian male physician who contracted Ebola virus disease (EVD) while working in an Ebola Treatment Unit in Sierra Leone. The course of infection was complicated by respiratory failure and *Plasmodium vivax* (*P. vivax*) co-infection.

## Case presentation

### Presenting concerns

A 50-year-old male physician contracted Ebola virus disease (EVD) while working in Sierra Leone. He had joined the Ebola treatment center on September 2014 and on November 20, he developed a single episode of vomiting, diarrhea and fever (37.5 °C). He had no history of previous malaria episode and a malaria antigen rapid test was negative. On November 24 he started artemisinine combined therapy, despite the negative rapid test for malaria. On this same day he tested positive to a molecular assay for Ebola virus (EBOV) and was medically evacuated to the Lazzaro Spallanzani National Institute for Infectious Diseases in Rome, by an Italian military flight [11]. At arrival (November 25) he was febrile and complained with severe fatigue but self-sufficient. He was admitted to a medical high level isolation unit specifically devoted for caring for patients with highly infectious diseases [12].

**Fig. 1:**
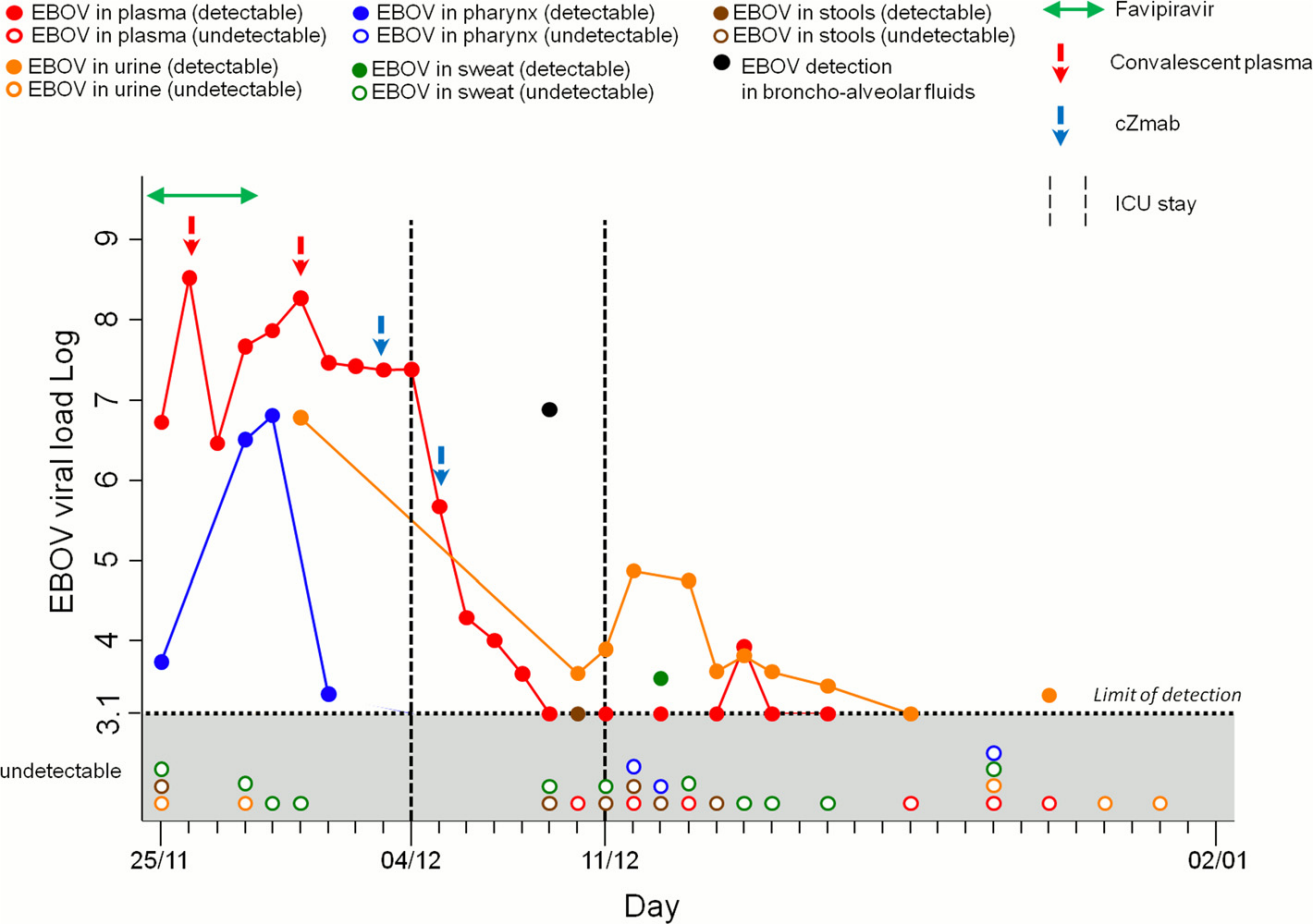
EBOV viral load in different clinical specimen and time of administration of experimental antiviral therapies

**Fig. 2:**
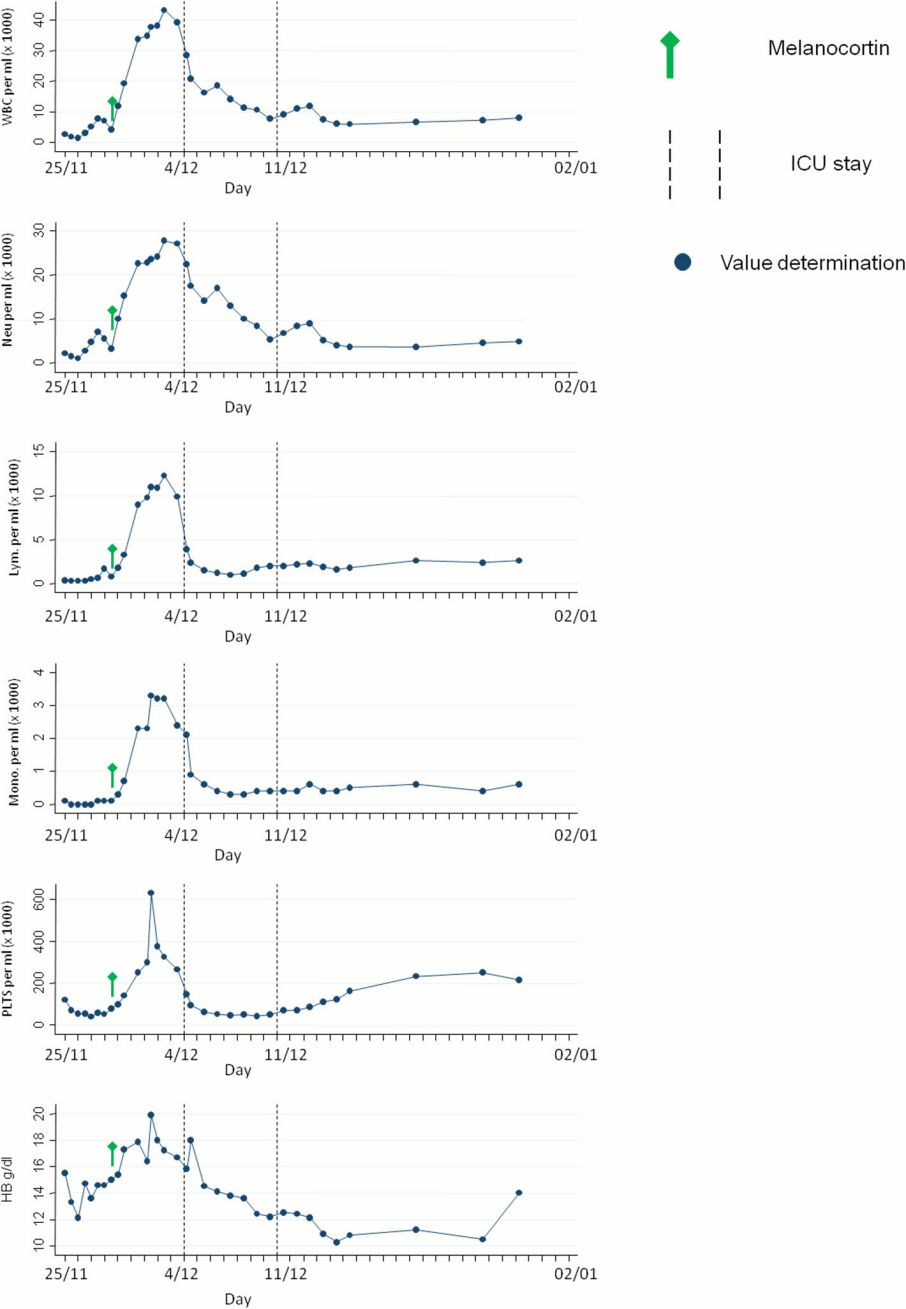
Blood counts during hospital stay. ICU= intensive care unit; WBC= white blood cells; Neu.= neutrophils; Lym.=lymphocytes; Mono.= Monocytes; PLTS= platelets; HB= hemoglobin

**Fig. 3:**
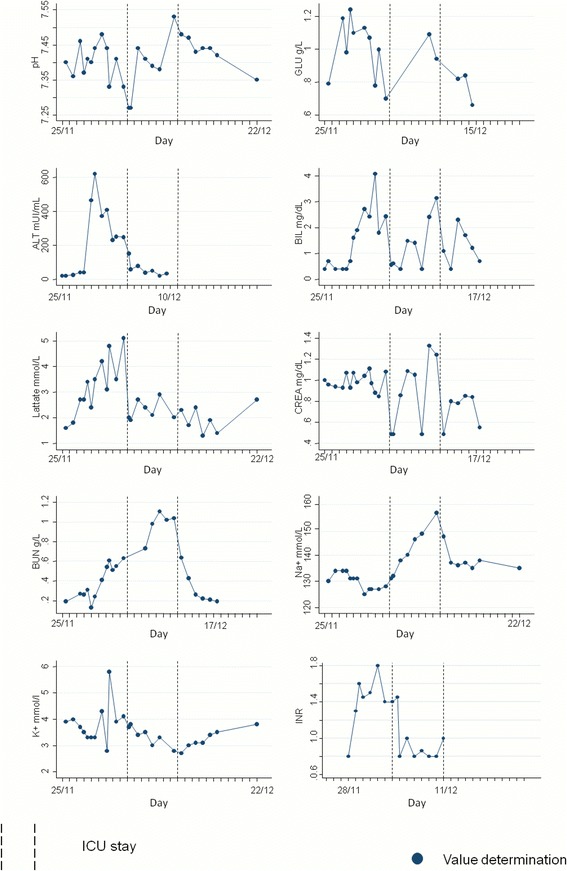
Clinical chemistry assays during hospital stay (all tests were performed on venous blood specimens). ICU= intensive care unit; GLU= glucose; ALT= alanine transaminase; BIL.= bilirubin; CREA.= creatinine; BUN.= blood urea nitrogen; Na+= sodium ; K+= potassium; INR= international normalized ratio

Table 1 reports patient’s signs and symptoms timeline. Figures 1, 2 and 3 reports EBOV viral load, blood counts and clinical chemistry, respectively. A detailed description of all laboratory assays is reported in Additional file 1. The timeline of patient’s clinical course is reported in Additional file 2.

**Table 1:** Patients clinical parameters during hospital stay

Day	Place of stay	Diarrhea a	fi02 (%)	S02 (%)	Fever (°C)	HR (beat/min)	Vomit (episode)	BP (mmHg)
25/11/2014	Medical	Yes	21	98	39.1	80	0	50/100
26/11/2014	Medical	Yes	50	95	38.4	89	0	80/120
27/11/2014	Medical	Yes	21	96	37.4	60	0	90/135
28/11/2014	Medical	Yes	21	97	39.4	131	8	70/120
29/11/2014	Medical	Yes	40	95	39.4	80	3	80/150
30/11/2014	Medical	Yes	40	95	39.1	70	3	90/145
01/12/2014	Medical	Yes	40	91	38.8	75	2	90/150
02/12/2014	Medical	Yes	50	90	39.4	80	2	80/140
03/12/2014	Medical	Yes	50	90 b	38.4	64	2	80/130
04/12/2014	ICU	Yes	60	87 c	37.8	70	2	80/130
05/12/2014	ICU	Yes	50	93 c	37.4	120	0	70/110
06/12/2014	ICU	Yes	40	91 c	39.2	110	0	70/105
07/12/2014	ICU	1300	40	92 c	37.8	105	0	70/115
08/12/2014	ICU	2100	30	93 c	36.5	95	0	65/110
09/12/2014	ICU	3300	30	93 d	37.5	100	0	75/120
10/12/2014	ICU	1700	35	92	38	95	0	85/130
11/12/2014	ICU	1500	31	94	37.8	95	0	80/120
12/12/2014	Medical	1700	35	97	38.1	110	2	90/150
13/12/2014	Medical	1000	35	98	37.4	110	1	80/110
14/12/2014	Medical	500	28	98	36.8	95	0	80/120
15/12/2014	Medical	300	28	99	36.5	90	0	70/110
16/12/2014	Medical	Na	21	98	36.6	90	0	85/130
17/12/2014	Medical	Na	21	98	36.0	85	0	80/120
18/12/2014	Medical	Na	21	98	36.3	80	0	70/110
19/12/2014	Medical	Na	21	98	36.9	78	0	80/120
20/12/2014	Medical	Na	21	98	36.5	75	0	80/120
21/12/2014	Medical	Na	21	98	37.0	70	0	70/110
22/12/2014	Medical	Na	21	98	36.4	70	0	90/130
23/12/2014	Medical	Na	21	98	36.6	70	0	80/120
24/12/2014	Medical	Na	21	98	36.5	70	0	75/110
25/12/2014	Medical	Na	21	98	36.5	70	0	80/120
26/12/2014	Medical	Na	21	98	36.5	70	0	70/130
27/12/2014	Medical	Na	21	98	36.4	70	0	80/130
28/12/2014	Medical	Na	21	98	36.5	70	0	90/130
29/12/2014	Medical	Na	21	98	36.4	70	0	70/120
30/12/2014	Medical	Na	21	98	36.5	70	0	80/110
31/12/2014	Medical	Na	21	98	36.5	70	0	90/130
01/01/2015	Medical	Na	21	98	36.5	70	0	80/120
02/01/2015	Medical	Na	21	98	36.5	70	0	70/110

a Diarrhea amount was provided in ml between 7 and 15 December while the rectal collector was in place; b: 66 % on air (Fi02 21 %); c: under mechanical ventilation; d: extubated; na: not any

### Clinical findings during hospital stay

#### Admission in medical isolation unit

**Fig. 4:**
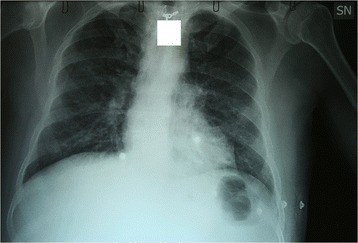
Chest XR carried out on December 2 2014. SN= left side; the white square was added for patient’s privacy protection

The patient remained in the medical isolation unit between November 25 and December 4, 2014. Patients’ clinical presentation was dominated by fever and gastrointestinal symptoms. At arrival he received fluid therapy, empiric antibiotic therapy and favipiravir. On November 26, therapy with convalescent plasma from one survivor of the present EBOV outbreak was given, the administration was followed by retrosternal chest pain, high fever, chills and low oxygen saturation resolving after hydrocortisone and clorfenamine administration. On November 28, a measles-like rash appeared on head, trunk and limbs and was accompanied by nausea and vomiting. On November 29, alanine amino transferase (ALT) concentration suddenly increased, while renal function and coagulation remained normal. Then we decided to suspend favivpiravir administration. November 30, the patient underwent a second administration of convalescent plasma from a different donor. Eventually patient received melanocortin [13] (a peptide for controlling “cytokine storms” and preventing vascular leakage syndrome) and steroids. The following day respiratory function gradually worsened and chest X-rays performed on December 2 showed bilateral interstitial pulmonary infiltrates (Fig. 4). On December 3, the patient received ZMAb [14, 15] a mixture of monoclonal antibody against EBOV glycoprotein. The administration was followed by a sharp reduction of the EBOV blood level. The same day due to progressive onset of severe respiratory failure the patient was moved to the intensive care unit (ICU).

#### Admission in ICU

The patient was cared for in the ICU between December 4 and 11. This unit meets requirement for high level bio-containment [12, 16]. Soon after admission, he underwent mechanical ventilation and a three-way central venous catheter (CVC) was inserted into the right internal jugular vein. A urinary catheter and a naso-gastric tube were also inserted.

While intubated, the patient underwent lung ultrasounds (Nanomaxx Sonosite®) according to Bedside Lung Ultrasound in Emergency (BLUE) protocol [17]. Pleural effusion and pneumothorax were ruled out, but the presence of up to four B-lines in all the standard points suggested a significant lung interstitial involvement. A progressive bilateral reduction in the number of B-lines, initially in the upper and lower BLUE points, and later in the posterolateral alveolar or pleural syndromes point, was observed during the ICU stay.

On December 5, he received a second dose of ZMAb followed by a second sharp decrease of EBOV viremia. On December 6, due to severe non-bloody diarrhea, a rectal fecal collector (Flexiseal® ConvaTec) was placed. On December 7, due to persisting fever another malaria test (PCR) was performed which yielded a positive results for *P. vivax* infection. Therefore anti-malaria therapy was started. On December 9, the patient underwent a bronchial aspirate; PCR on bronchial aspirate fluids evidenced a viral load of 6.88 Log copies of EBOV RNA/ml. All PCRs for other viral and bacterial respiratory pathogens were negative (see Additional file 1). On December 10, the patient was extubated and moved to the medical isolation unit one day later.

#### Recovery and discharge

After readmission to the medical isolation unit the clinical conditions gradually improved. On December 11, all antimicrobials were suspended considering the improvement of general conditions and persistent lack of positive cultural and molecular test for bacteria and fungi (Additional file 1). Since December 14 fever completely disappeared. On December 17, CVC, urinary catheter and rectal fecal collector were also removed. On December 21, the patient completely recovered.

A progressive increase of the anti-EBOV IgM titer was observed from December 11, reaching a peak of 1:160 on December 25. The anti-EBOV IgG titer was stably at 1:160 until December 25, presumably as the result of convalescent plasma administration, and eventually increased until discharge. The patients was discharged on January 2 after that two consecutive negative EBOV PCR assay results from blood, urine, sweat (axillary swab) and stool were obtained (see Fig. 1).

### Therapeutic intervention

#### General supportive therapy

The patient received 4-5 L/day of intravenous hydration with crystalloid solution since admission for 18 days. The amount and composition of the solution were based on the daily plasma determination of electrolytes, venous pH and daily fluid output. Between November 28 and December 4, he received parenteral nutrition and, eventually, enteral nutrition between December 5 and 11 (approximately 2000 kCal/day). Between December 4 and 11, he received norepinephrine (0.05 mcg/kg/min) and furosemide (20 mg tid).

#### Antimicrobials

The patient received empiric antibiotic treatment for 18 days since admission. On November 25, he started ceftriaxone (2000 mg daily) and oral levofloxacin (500 mg bid). On November 28, ceftriaxone and levofloxacin were stopped due to the onset of severe diarrhea and increase of liver enzymes. Meropenem (1000 mg tid i.v.) plus metronidazole (500 mg qid i.v.) were started. On December 4, due to the persistence of fever and diarrhea, meropenem and metronidazole were discontinued and anidulafungin (200 mg as a loading dose and then 100 mg daily i.v.), piperacillin/tazobactam (4500 mg qid i.v.), linezolid (600 mg bid i.v.) and oral vancomycin (125 mg qid) were started. Oral vancomycin was discontinued after a negative *Clostridium difficile* PCR assay on stools. On December 9, piperacillin/tazobactam and linezolid were discontinued, and metronidazole (500 mg qid i.v.) was started.

#### Anti-malarial therapy

Anti-malarial therapy with chloroquine (600 mg time 0 and then 300 mg after 6, 24 and 48 h) was given through a nasogastric tube. Primaquine was prescribed for future use after G6PDH determination following discharge.

#### Melanocortin

Melanocortin (TCS 10) [13] was administered to prevent plasma leakage syndrome; two 10 mg i.v. bolus 1 h apart from each other, followed by a 6 h continuous infusion of 50 mg in 60 ml of 0.9 % NaCl solution.

#### Favipiravir

The oral RNA polymerase inhibitor favipiravir [18–20] was administered orally for four days: loading dose in 3 fractionated doses (2400 mg followed by 2400 mg and then 1200 mg), then 1200 mg bid (cumulative dose 9,600 mg).

#### Convalescent plasma

The first course of convalescent plasma consisted in a 250 ml unit of a compatible blood group plasma to be administered with paracetamol and clorfenamine premedication. Due to insurgence of an adverse reaction oxygen therapy (50 % Fi O2) and 500 mg hydrocortisone plus clorfenamine 10 mg were given i.v. infusion for 2 subsequent days.

The second course of convalescent plasma consisted in a 250 ml unit of a compatible blood group plasma from a different donor that was administered after premedication with paracetamol, clorfenamine and 500 mg metylprednisolone. In this case no adverse reaction was reported.

#### ZMAb

Monoclonal antibodies were diluted, filtered with a 0.22 μm filter and topped with 0.9 % NaCl solution to a final volume of 1000 ml. After premedication with 1 mg/kg methyl prednisolone, ZMAb was infused in 12-15 h via a peripheral line. No adverse reactions were observed.

## Discussion

We reported the case of a 50-year-old Italian physician with severe EVD due to EBOV Makona variant, complicated with severe interstitial pneumonia and *P. vivax* co-infection. He received prompt fluid replacement and electrolyte supplementation to prevent volume depletion, metabolic disorders and hypovolemic shock [21, 22]. He was finally discharged in good clinical conditions 39 days after admission.

Upon admission, the patient presented with severe lymphopenia and moderate low platelet count compatible with EBOV infection [23] and P*. vivax* co-infection. On day 5 after admission, a sudden and sharp increase of WBC and platelets occurred, likely due to exogenous steroid administration and to melanocortin-induced endogenous glucocorticoid production [24].

Severe interstitial lung involvement was the main complication. Chest X-rays, lung ultrasounds and the high EBOV RNA concentration in the bronchial aspirate were strongly suggestive of EBOV-related interstitial pneumonia. Other authors reported respiratory failure in EVD possibly due to vascular leakage [7] and transfusion related acute lung injury (TRALI) [8]. However, we believe that vascular leakage was unlikely in this case because of the lack of both multi-organ involvement and coagulation abnormalities, that characterize vascular leakage syndrome [7, 25]. In addition the presence of high EBOV RNA load in the lower respiratory tract secretions, in the absence of detection of other common respiratory pathogens, supports the hypothesis that EBOV could have directly contributed to the lung damage. In fact, it is unlikely that the EBOV RNA detected in bronchial aspirate fluids could represent a trivial result of spill over from the blood compartment, eventually accompanied by delayed clearance, since the concomitant blood sample showed barely detectable EBOV RNA, and the blood concentration in the previous 3 days was 2.58-3.29 Log lower than the concentration in the bronchial aspirate. The most likely explanation for these findings is that the virus actually replicated into the lower respiratory tract. Noteworthy, direct viral involvement in lung injury has been already suggested in patients who received care in the USA [10]. Moreover, the hypothesis that the EBOV can actually replicate into lungs is supported by a recently published review which collect results of post mortem examinations from 89 cases of fatal filoviral infections in humans (24 of which with EBOV). This analysis provides evidence for viral replication in lungs by demonstrating viral antigens in several lung tissues and the presence of EBOV nucleic acids in resident macrophages [26]. Whether the *P. vivax* co-infection might have contributed to the lung injury can be hardly ascertained; however, acute respiratory distress syndrome is a frequent clinical feature in the rare circumstance of fatal *P.vivax* infection [27].

In our opinion, mechanical ventilation was crucial for survival in our patient because supported respiratory function and prevented muscular exhaustion, which allowed the patient to recover from the acute lung injury. This underlines that prompt access to advanced life support provides a substantial clinical benefit in EVD [28].

Despite 3 negative malaria antigen assays, the patient eventually tested positive in a PCR assay for *P. vivax*. Malaria co-infection was recently reported in an EVD patient who received care in the USA [9]. Our experience highlights that malaria antigen assays may offer an incomplete diagnostic picture leading to sub-optimal clinical management. Consequently, patients with EVD returning from areas endemic for malaria should receive empirical malaria treatment if neither molecular or microscopy assays are available or if concerns on malaria are raised.

Our patient also received empirical therapy against bacteria, fungi and intestinal protozoa. Severe bacterial super-infection has already been reported in subjects who received care for EVD in Europe [6]. At present, there is no definitive evidence to predict the actual risk of microbial super-infection in EVD patients. Nevertheless, we feel that the use of anti-infective drugs is a prudent approach. In fact, EVD patients may develop severe infective complications as the consequence of bacteria translocation from the gut [6], from equipment used for parenteral nutrition [29] or co-infection with tropical intestinal protozoa [30].

Our patient received three different experimental antiviral treatment, including favipiravir, convalescent plasma and ZMAb.

Favipiravir was given for 4 days. During this time, the patient experienced worsening of gastrointestinal symptoms, and an increased of ALT and bilirubin levels. This report provides no evidence of any causative association between favipiravir and either potential adverse events or viral load variation. In particular, gastrointestinal and liver abnormalities may be the consequence of *P. vivax* and EBOV infections. In fact, data from uncontrolled studies in EBOV-confirmed that patients did not report significant hepatic or gastrointestinal toxicity for similar favipiravir dosages but fail in providing any conclusive evidence on drugs efficacy [31, 32].

The two administrations of convalescent plasma were associated with a decrease viral load of 2.10 and 0.80 Log RNA copies/ml, respectively. The adverse event after the first plasma administration may have been due to an immune reaction to factors of the unknown donor and/or the lack of premedication with steroids. The possible influence of such adverse reaction on lung injury cannot be ruled out. Nevertheless, transfusion related acute lung injury (TRALI) could have hardly had a primary role. In fact, TRALI is an acute event which occurs within 6 h after transfusion [33]. In contrast our patients had lung function significantly worsened only since December 3, this is 7 and 3 days after the first and second convalescent plasma transfusion, respectively. Beside the two plasma donors (one of whom was a female) had no history of blood transfusions or previous pregnancies, which reduce possibility of TRALI [33].

Administration of ZMAb [14, 15] was temporally associated with a sharp and sustained decay of plasma viral load. As the increase of IgM and IgG was observed after the start of viremia decay, it is unlikely that the endogenous antibody response could be responsible for the observed decay of EBOV RNA, although the administration of convalescent plasma is a major confounder. The potential beneficial effect of monoclonal antibody therapy against EVD was reported in a recent experience describing the clinical management of few EVD patients treated with ZMapp (a similar, though not identical compound) [9, 10]. Interestingly, monoclonal antibody therapy against EBOV is one of only two clinical interventions capable of decreasing viral loads and significantly reduce the mortality of non-human primates infected with EBOV in controlled conditions [34]. Although, no causal association between administration of ZMAb and the patient’s outcome can be inferred; in fact, the viral decay load might have been coincidental rather than due to the ZMAb administration.

## Conclusion

This report describes the successful management of a patient with severe EVD in a setting with high healthcare standards. Several issues emerge from our experience. Firstly, fluid replacement and advanced life support with mechanical ventilation were pivotal for the patient’s recovery, and in our opinion, all patients with EVD should have access to this level of care. Secondly, it is prudent to start empirical therapy for malaria in the absence of reliable testing, such as PCR assays. Finally, similarly to other individual experiences, we observed a close temporal association between administration of ZMAb and viral load decay, suggesting that clinical and translational research on these new promising molecules should be prioritize.

## Consent

Written informed consent was obtained from the patient for publication of this Case report and any accompanying images.

The INMI’s Institutional Ethical Board assessed the criteria for access to experimental drugs and invasive procedures, approved informed consent form and analyzed ethical issues and possible solutions to minimize the physical and psychological harm for the patient. The patient signed an informed consent for any single procedure or treatment performed, after thoroughly explanation of reasonably anticipated benefits and potential hazards of intervention.

Emergency Use Authorization for investigational new drugs was issued by the Italian Drug Agency (AIFA), the authority entitled to approve medical agents to be used for therapy of disease when they are not the standard of care or supported by research that proves their safety.

## Additional files

Additional file 1:
**Laboratory methods.**
Additional file 2:
**Clinical course timeline.**
Additional file 3:
**Person and institution involved in the organization and management of the patient.**

## Abbreviations

EBOV: Ebola virus Makona variant
EVD: Ebola virus disease
PCR: Polymerase chain reaction
RNA: Ribonucleic acid
*P. vivax*: *Plasmodium vivax*
ALT: Alanine amino transferase
ICU: Intensive care unit
CVC: Central venous catheter
mg: Milligrams
ml: Milliliters
tid: Ter in die (thrice time a day)
bid: Bis in die (twice a day)
qid: Quater in die (four times a day)
i.v.: Intravebeous
Log: Decimal logarithm
