# Supplementary material for: Ebola virus disease complicated with viral interstitial pneumonia: a case report
Source: BMC Infect Dis. 2015 Oct 16;15:432. doi: 10.1186/s12879-015-1169-4 (PMC4608352; doi:10.1186/s12879-015-1169-4)
Supplement: Additional file 1: — Laboratory methods. [file 12879_2015_1169_MOESM1_ESM.docx]

# Additional file 1: Laboratory methods

## Hematochemistry laboratory assays

Blood glucose, creatinine, ALT, amylase, potassium and lattate were assessed by Reflotron Plus Clinical Chemistry Analyser (Roche   Diagnostics, Rotkreuz, Switzerland). Gas analyses was carried out by GEM Premier 3000 (Werfen, Barcelona, Spain). ABX Micros ES 60 Hematology analyzer (Horiba,  [Kyoto](https://www.google.it/search?biw=1034&bih=595&q=kyoto+giappone&stick=H4sIAAAAAAAAAGOovnz8BQMDgwsHnxCXfq6-gVFhTkpGshIHiG2ZYhKvpZWdbKWfX5SemJdZlViSmZ-HwrHKSE1MKSxNLCpJLSqOOJkZvTlCdPKX33PenTroeLqzquEkAGF2dBRhAAAA&sa=X&ei=paTDVJ_6GMi9Udi0grAB&ved=0CJkBEJsTKAEwEQ), Japan) was used for complete blood count. International normalized ratio (INR) was assessed by Hemochron® Signature Plus Microcoagulation system (Cremascoli & Iris).

## Microbiological assays

Potential coinfection with Malaria was assessed through antigen detection BinaxNOW® Malaria test (Alere Inc, Scarborough, ME, USA) and with in-house specific PCR.[^[[1]](#endnote-1)^]

Dengue virus NS1 Ag + IgG/IgM detection was performed with the rapid POCT SD BIOLINE Dengue Duo (Standard Diagnostics Inc. Gyeonggi-do, Republic of Korea).

Blood cultures were performed by non-automated procedure using Hemoline DUO blood culture bottles (Biomerieux, Craponne, France).

Presence of microbial DNA was assesses on nucleic acids extracted with the automated QIAcube system (Qiagen, Hilden, Germany), using the QIAamp DNA mini kit for EDTA-blood and bronchial aspirate, and Stool DNA mini kit for stool samples.

In house methods were used to perform PCR for bacterial 16S on DNA extracted from blood [^[[2]](#endnote-2)^], for Salmonella spp. [^[[3]](#endnote-3)^] and for *E.* *histolytica* and *E. dispar* [^[[4]](#endnote-4)^] on DNA extracted from stool samples.

GenoType EHEC (v2.0) kit (HAIN Lifescience GmbH, Nehren, Germany) was used to identify Enterohaemorrhagic, enteropathogenic and enteroinvasive *E. coli*, and *Shigella spp*. genomes in DNA extracted from stool samples.

C.Diff Quik Chek Complete Assay (Techlab-Alere Inc, Scarborough, ME, USA) was used to identify *C. difficile genomes in*  DNA extracted from of stool samples

The Seeplex®PneumoBacter ACE Detection (v3.0) multiplex-PCR kit (Seegene, Eschborn, Germany) was used to identify respiratory pathogens ( *S. pneumoniae, H. Influenzae, C. pneumoniae, L. pneumophila, B. pertussis, M. pneumoniae)* in DNA extracted from bronchial aspirate fluids.

Detection of Influenza A and B viruses was performed with in house real time PCR assays established according to Centers for Diseases Control and Prevention (CDC) for influenza A, and WHO for influenza B. [^[[5]](#endnote-5)^]

Other respiratory viruses (ADV, Boca, MPV, RSV A/B, Para 1,2,3,4, Rino/Entero, HCoV) were investigated using a commercial multiplex PCR kit (Respiratory Multi Well System R-gene, Argene, Verniolle, France).

The occurrence of common respiratory virus infection was also investigated by testing serum antibodies by Complement Fixation (CF) using a kit from a standard Kolmer microthecnique. The occurrence of neutralizing and complement fixing antibodies in rubella on serum samples collected on the day of discharge and after one month. Standard reagents for the development of the CF reaction were from commercial source (Institut Virion/Serion GmbH, Germany). Serial serum dilutions (1:10 up to 1:80) were tested. Negative and high-positive controls were included .

## Ebola diagnostics

EBOV RNA in plasma was tested using Real Star Filovirus Screen RT-PCR 1.0 kit (Altona Diagnostics, Hamburg, Germany). Viral genome quantification was based on a standard reference curve provided by the kit producers. In addition to plasma, Ebola molecular detection was carried out also in urine, mucosal and cutaneous swabs, stools and respiratory samples. Confirmation of EBOV real time PCR result and preliminary viral characterization were carried out by ABI Prism 310 Genetic Analyizer (Applied Biosystems, Forster City, CA, USA), using the BigDye Terminator Cycle Sequencing Kit (Applied Biosystems) as already reported by Panning at al.[^[[6]](#endnote-6)^] and Ogawa H et al.[^[[7]](#endnote-7)^] Viral sequences were aligned using CLUSTALW X1.5 software (http://npsa-pbil.ibcp.fr/) and were registered in the GenBank nucleotide database (accession numbers KP231870.1 and KP339863.1)**.**

The presence of EBOV-specific IgG and IgM antibodies was determined by means of in house indirect immunofluorescence assay, using slides prepared with a mixture of EBOV- infected and uninfected Vero E6 cells.

EBOV was isolated on Vero E6 cell cultures from plasma collected immediately after admission (EBOV RNA: 5,43E+06 copies/ml). Virus isolation from plasma collected on day 25 (EBOV RNA detected <Limit of Detection-LOD, 1.3+03 copies/ml), from urine samples collected on day 20 (EBOV RNA 5,71E+04 copies/ml) and 21 (EBOV RNA 4,29E+03 copies/ml), and from sweat (axillary swab) collected on day 19 (EBOV RNA 3,43E+03 copies/ml) resulted negative after four blind passages.

## Reference

1. Snounou G, Viriyakosol S, Zhu XP, Jarra W, Pinheiro L, do Rosario VE, Thaithong S, Brown KN. High sensitivity of detection of human malaria parasites by the use of nested polymerase chain reaction. Mol Biochem Parasitol. 1993 Oct;61(2):315-20. [↑](#endnote-ref-1)
2. Richardson DC, Louie L, Louie M, Simor AE. Evaluation of a rapid PCR assay for diagnosis of meningococcal meningitis. J Clin Microbiol. 2003 Aug;41(8):3851- [↑](#endnote-ref-2)
3. Nair S, Lin TK, Pang T, Altwegg M. Characterization of Salmonella serovars by PCR-single-strand conformation polymorphism analysis. J Clin Microbiol. 2002 Jul;40(7):2346-51 [↑](#endnote-ref-3)
4. Evangelopoulos, A., Spanakos, G., Patsoula, E.,Vakalis, N., Legakis, N., 2000. A nested, multiplex, PCR assay for the simultaneous detection and differentiation of Entamoeba histolytica and Entamoeba dispar in faeces. Ann. Trop. Med. Parasitol. 94, 233–240. [↑](#endnote-ref-4)
5. http://www.who.int/csr/resources/publications/swineflu/realtimeptpcr/en/index.html) [↑](#endnote-ref-5)
6. Panning M, Laue T, Olschlager S, Eickmann M, Becker S, Raith S, Courbot MC, Nilsson M, Gopal R, Lundkvist A, di Caro A, Brown D, Meyer H, Lloyd G, Kummerer BM, Gunther S, Drosten C. Diagnostic reverse-transcription polymerase chain reaction kit for filoviruses based on the strain collections of all European biosafety level 4 laboratories. J Infect Dis. 2007 Nov 15;196 Suppl 2:S199-204 [↑](#endnote-ref-6)
7. Ogawa H, Miyamoto H, Ebihara H, Ito K, Morikawa S, Feldmann H, Takada A. Detection of all known filovirus species by reverse transcription-polymerase chain reaction using a primer set specific for the viral nucleoprotein gene. J Virol Methods. 2011 Jan;171(1):310-3. [↑](#endnote-ref-7)
