# Supplementary figures and images for: Ebola virus disease complicated with viral interstitial pneumonia: a case report
Source: BMC Infect Dis. 2015 Oct 16;15:432. doi: 10.1186/s12879-015-1169-4 (PMC4608352; doi:10.1186/s12879-015-1169-4)

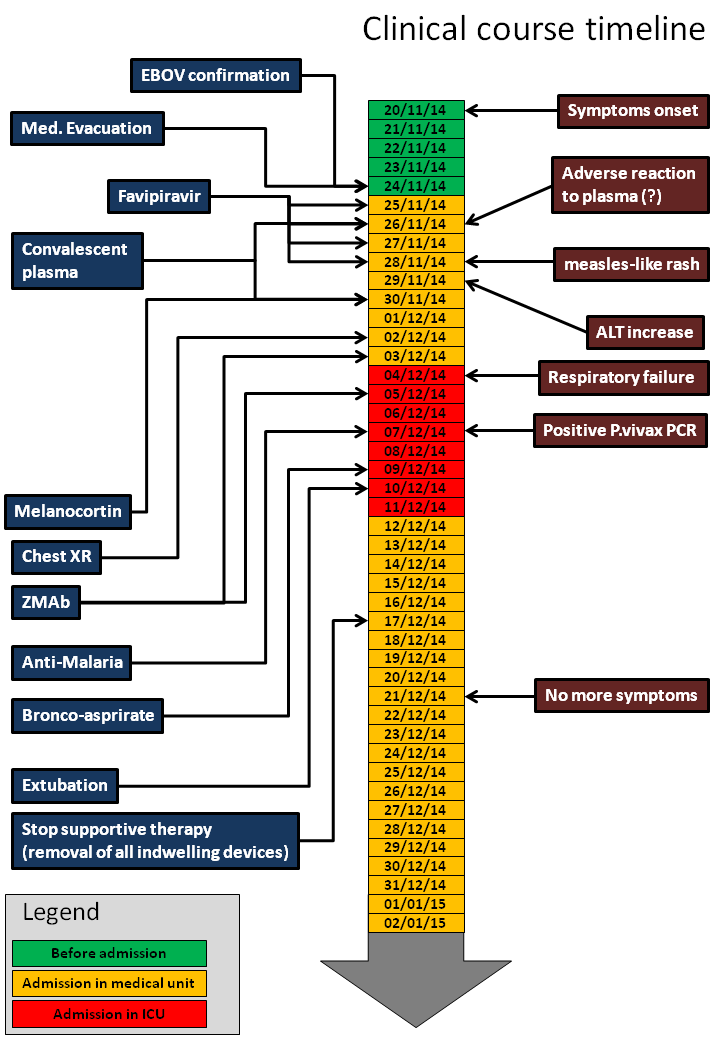

Supplement: Additional file 2: — Clinical course timeline. [file 12879_2015_1169_MOESM2_ESM.gif]
