# Supplementary material for: Ebola virus disease complicated with viral interstitial pneumonia: a case report
Source: BMC Infect Dis. 2015 Oct 16;15:432. doi: 10.1186/s12879-015-1169-4 (PMC4608352; doi:10.1186/s12879-015-1169-4)
Supplement: Additional file 3: — Person and institution involved in the organization and management of the patient. [file 12879_2015_1169_MOESM3_ESM.docx]

**Additional file 3: person and institution involved in the organization and management of the patient**

**INMI’s Institutional Independent Ethical Board**

- Mauro Moroni, Cinzia Caporale, Alberto Chiriatti, Giulio Maria Corbelli, Antonio Cristaudo, Fabio Francario, Caterina di Viggiano, Paolo Oliva, Fabrizio Palmieri, Patrizio Pezzotti, Luca Steardo, Giuseppe Ippolito, Andrea Antinori, Silvia Murachelli, Enrico Girardi, Cristina Costa

**Italian Institutions**

- Ministry of Health of Italy: Minister (Beatrice Lorenzin), Chief of the Cabinet (Giuseppe Chinè), Diplomatic Adviser to the Minister (Luigi Ferrari), Director General for health prevention (Ranieri Guerra), Directorate for health prevention infectious diseases and board health control offices (Maria Grazia Pompa, Loredana Vellucci and their staff), USMAF Fiumicino (Francesco Paolo Maraglino e the staff) e USMAF Malpensa (Mario Germagnoli and the staff), Spokesperson of the Minister (Claudio Rizza), Chief of Press Office (Fabio Mazzeo), Press Office (Consuelo Gasperini),
- Italian Presidency of Council – Office of Humanitarian and Governmental flights (Giovanni Bardelli)
- Ministry of foreign affairs, Crisis Unit (Claudio Taffuri, Edoardo Pucci)
- Italian Air Force (Roberto Biselli, Piervalerio Manfroni, Pasquale Preziosa and all the staff involved in the medical evacuation from Sierra Leone to Italy)
- National Blood Center (Giuliano Grazzini)
- National Drug Agency- AIFA (Marra Anna Rosa Marra, Sandra Petraglia, Maria Paola Trotta, Patrizia Marconi, Francesca Mastroianni, Luca Pani)
- Customhouses of Fiumicino and Malpensa
- The Polizia di Stato who made the escort during the transport of the patient and of the drugs
- The fire department and Carabinieri who escorted the transport of patient
- The Italian Red Cross for the participation in the transport
- The Department of Civil Protection (Marco Rinaldelli, Laura Trovalusci, Alessandro Bianco) supported by Damiano Travaglini (Regional Blood Center)
- The staff of blood center at S.Camillo hospital (Michela Miceli, Marco Possenti, Barbara Rizzoli, Alessandro De Rosa)
- The Lazio Region, the President (Nicola Zingaretti) General Director for Health (Flori Degrassi), General Directorate for Health (Amalia Vitagliano), Director of the Control Room (Alessio D’Amato)

**International Institutions**

- World Health Organization WHO-Geneva Switzerland - Nahoko Shindo (Lead, Ebola Clinical Standards), Nicola Magrini (Department of Essential Medicines), Maurizio Barbeschi (Global Capacity, Alert and Response)
- European Commission, DG SANCO (Michel Pletschette, Paolo Guglielmetti, John Ryan)
- Special Pathogens Program, Public Health Agency of Canada, Winnipeg, Manitoba, Canada (Gary Kobinger, Xiangguo Qiu)
- Centers for Disease Control and Prevention, Atlanta, GA, USA (Timothy Uyeki)
- Bernhard Nocht Institute for tropical medicine, Section Parasitology (Egbert Tannich)
- University Hospital Frankfurt, Frankfurt/Main, Germany. Infectious Diseases Unit (Timo Wolf), Anaesthesiology, Intensive Care Medicine (Kai Zacharowski), blood center (Richard Schäfer)
- University Hospitals of Geneva, Switzerland, Intensive Care (Jérôme Pugin), Infectious Diseases/Virology (Laurent Kaiser, Pauline Vetter)
- Hospital La Paz, Madrid, Spain: Infectious Diseases Unit, Internal Medicine Service (José R Arribas), Blood Center (Aurora Viejo, Mercedes Gazquez Juarez)
- Instituto de Salud Carlos III, Madrid, Spain (María Paz Sanchez-Seco, Pedro Anda)
- Agence Nationale de Sécurité du Médicament et des produits de santé-ANSM (Alban Dhanani)
- Etablissement de Préparation et de Réponse aux Urgences Sanitaires – EPRUS (French Establishment for the Preparation and Response to Health Emergencies), La plaine Saint Denis, France (Lionel de Moissy, Frédéric Grelet)
- IHU Mediterranee Infection,Southern France Referral Center for Ebola Care, Marseille, France (Philippe Brouqui)
- Service de Maladies Infectieuses et Tropicales, Hôpital Bichat Claude Bernard, Paris, France, (Yazdan Yazdanpanah)
- Agencia Española de Medicamentos y Productos Sanitarios (AEMPS) Madrid-Spain (César Hernàndez Garcìa)
- Ministerio de Sanidad, Servicios Sociales e Igualdad, Madrid-Spain (Karoline Fernández de Hoz Zeitler)
- Embassy of Japan in Rome (Yuki Yamaguchi)

**Industrial research, diagnostic, pharmaceutical and regulatory companies**

- Altona Diagnostics GmbH. Hamburg, Germany (Stephan Ölschläger)
- Chimerix Ltd. Durham North Carolina- USA (Garreth Nichols, Michelle Berrey)
- Metis Clinical UK (Allison Morgan)
- Optum- Regulatory Services, Life Sciences Orlando, Florida (Silvia Bendiner)
- Toyama Chemical Co., Ltd./Fuji Chemical Industry Co., Ltd. Japan (Hiroshi Kitaguchi, Satoru Uriya)
- Medivector Boston, MA, USA (Carol R. Epstein)
- Mapp Biopharmaceutical, Inc. San Diego, CA USA (Tara Nyhuis, Larry Zeitlin)
- Health Ricerca e Sviluppo - HR&S, Massa Lombarda, RA, Italy (Giorgio Noera, Claudio Camerino)
- MChE-F4Pharma, Vienna, Austria (Thomas Steiner)
- Accella Advisors GmbH, Uetliburg SG, Switzerland (Petra Wülfroth)

**Couriers**

The staff of the couriers who performed a participative delivery of drugs and plasma PHSE, DHL (Carlo De Ruvo), World Courier, Federal Express and especially for PHSE (Emmanuela Cannizzaro, Gianluca Meneguzzi)

**Contractors operating at INMI**

• The staff of Sameco for transport and dispose of special waste

• The staff of Manutencoop for cleaning (Marco Tuminiello)

• The staff of security companies (Città di Roma Metronotte, Roma Union Security) for control of access to high biocontainment isolation and laboratories areas

**Others**

Emergency (Gina Portella, medical coordinator, Lakka Ebola Treatment Unit, Sierra Leone)
